# Expanding the Activity of Tissue Inhibitors of Metalloproteinase (TIMP)-1 against Surface-Anchored Metalloproteinases by the Replacement of Its C-Terminal Domain: Implications for Anti-Cancer Effects

**DOI:** 10.1371/journal.pone.0136384

**Published:** 2015-08-26

**Authors:** Jing Xian Duan, Magdalini Rapti, Anastasia Tsigkou, Meng Huee Lee

**Affiliations:** 1 From the Department of Biological Sciences, Xian Jiaotong Liverpool University, 111 Ren Ai Road, Suzhou, China; 2 Department of Oncology, Cambridge University, Cancer Research Institute, Cambridge, United Kingdom; University of Patras, GREECE

## Abstract

Tissue inhibitors of metalloproteinases (TIMPs) are the endogenous inhibitors of the matrix metalloproteinases (MMPs) and a disintegrin and metalloproteinases (ADAMs). TIMP molecules are made up of two domains: an N-terminal domain that associates with the catalytic cleft of the metalloproteinases (MP) and a smaller C-terminal domain whose role in MP association is still poorly understood. This work is aimed at investigating the role of the C-terminal domain in MP selectivity. In this study, we replaced the C-terminal domain of TIMP-1 with those of TIMP-2, -3 and -4 to create a series of “T1:TX” chimeras. The affinity of the chimeras against ADAM10, ADAM17, MMP14 and MMP19 was investigated. We can show that replacement of the C-terminal domain by those of other TIMPs dramatically increased the affinity of TIMP-1 for some MPs. Furthermore, the chimeras were able to suppress TNF-α and HB-EGF shedding in cell-based setting. Unlike TIMP-1, T1:TX chimeras had no growth-promoting activity. Instead, the chimeras were able to inhibit cell migration and development in several cancer cell lines. Our findings have broadened the prospect of TIMPs as cancer therapeutics. The approach could form the basis of a new strategy for future TIMP engineering.

## Introduction

Matrix metalloproteinases (MMPs) and a disintegrin and metalloproteinases (ADAMs) are members of the zinc-dependent metzincin super-family. There are at least twenty-three known MMPs and twenty one ADAMs identified in human but not all the ADAMs are enzymatically active. MMPs are multi-domain enzymes with a defined domain organisation. At the N-terminus of their sequences is a pro-domain that contains a cysteine switch that keeps the enzyme in dormant form until activated. Succeeding the pro-domain is a highly conserved catalytic domain in which the zinc-binding motif HExxHxxGxxH resides. With the exception of the matrilysins (MMP7 and -26), the MMPs contain a four-bladed, propeller-shaped haemopexin domain downstream of the catalytic domain. The majority of the MMPs are secreted, only a few are tethered to the cell surface either via a transmembrane domain (MMP14, -15, -16 and -24) or glycosylphosphatidylinositol (GPI) anchor (MMP-17, -25). The MMPs are important regulators of the extracellular milieu as the enzymes degrade components of the extracellular matrix (ECM) such as collagens, laminin, chondroitin sulphate proteoglycans as well as release a range of cytokines, growth factors and their receptors including E-cadherin, ephrin, HB-EGF, amphiregulin, TGF-β and Fas ligand (reviewed in [1–4]).

Often called the “sheddases”, ADAMs are all type I transmembrane proteinases. Structure-wise, ADAMs consist of a pro-domain, a metalloprotease domain, a disintegrin domain, a cysteine-rich/EGF-like domain followed by a transmembrane anchor and an intracellular cytoplasmic tail. The involvement of the ADAM proteinases, in particular ADAM10 and -17 (a.k.a. TNF-α converting enzyme, TACE) in the release of pro-inflammatory cytokines such as TNF-α and IL-6 has rendered the ADAMs prime targets for drug discovery in arthritis and cancers [5, 6]. In concert, MMPs and ADAMs regulate cellular microenvironment through modulation of the ECM components and release of bioactive molecules essential for cell growth and development. ADAM17 and MMP14 are particularly well known for their ability to promote cancer progression [5–8]. Down regulation of the proteases by either gene silencing or hydroxamate inhibitors have been shown to be an effective means of blocking cancer metastasis *in vivo* [9–12].

The enzymatic activity of the MMPs and ADAMs are modulated by the endogenous inhibitors, tissue inhibitors of metalloproteinases (TIMPs). TIMPs are all small proteins of approximately 24 kDa in mass. Crystallographic and NMR studies show that TIMP molecules are comprised of two functional domains: an N-terminal domain of approximately 15 kDa that folds into an oligonucleotide/oligosaccharide-binding motif and a structurally less well defined 8 kDa C-terminal domain composed largely of β-sheets [13, 14]. TIMPs inhibit the metalloproteinases (MP) by inserting their “MMP-binding ridges” into the catalytic cleft of the proteinases to form a 1:1 stoichiometric enzyme-inhibitor complex. There are four human TIMPs (TIMP-1 to -4), each TIMP has its own distinctive profile of MP selectivity. MMP14, for instance, is sensitive to TIMP-2, -3 and -4 but not TIMP-1 [15]. ADAM17, in contrast, is selectively inhibited by TIMP-3 [16].

The role of TIMP-1 in tumorigenesis has been a rather controversial one. On the one hand, it is a well-documented fact that TIMP-1 inhibits MPs involved in bioactive molecule shedding and ECM turnover and by so doing, suppresses tumor development [17–19]. On the other hand, TIMP-1 also possesses non MP-related cell stimulating and pro-angiogenesis functions that have since precluded its development as a viable therapeutic agent against cancers (reviewed in [20, 21]). Effects to engineer the TIMPs against the MPs have so far focused on the N-terminus of the molecules due to the ease of production of the domain. Much less is known about how the C-terminal domain interacts with the MPs as the domain cannot be produced as an independent entity. In this study, we demonstrate that the C-terminal domain can also be exploited for engineering. Indeed, the activity of TIMP-1 can be significantly expanded against ADAM10, ADAM17, MMP14 and MMP19 by the replacement of its C-terminal domain.

## Materials and Methods

### Materials

ADAM10, MMP13 and the fluorogenic peptide substrate IX ((7-Methoxycoumarin-4-yl)acetyl-Lys-Pro-Leu-Gly-Leu-N-3-(2,4-Dinitrophenyl)-L-2,3-diaminopropionyl-Ala-Arg-NH_2_) were products of R&D Systems, MN. ADAM17 (residue 1–473), MMP14 and MMP19 were produced in house using Sf-9 insect cell or refolded from *Escherichia coli* inclusion bodies following published protocols [15, 22, 23]. Nickel sepharose excel resin was obtained from GE Healthcare Life Sciences, USA. Human TNF-α ELISA kit was a product of Sino-Biologic Inc., China. Mammalian cell lines were purchased from the Shanghai Cell Bank, Chinese Academy of Science.

### T1:TX chimera construction, protein production and purification

The process of making N-TIMP-1:C-TIMP-2/-3/-4 (named “T1:TXs” hereafter) chimeras has been described previously [24]. For protein expression, Sf-9 cell (1.5 x 10^6^ ml^-1^) was infected with baculovirus (MOI 1:1) for four days before protein extraction with 0.5 ml nickel sepharose excel resin. Adsorption was allowed for 2 hours before the nickel resin was harvested and washed with 10 ml wash buffer (50 mM Tris HCl pH 7.8, 250 mM NaCl, 5% glycerol and 20 mM imidazole) followed by elution with 3 ml elution buffer (50 mM Tris HCl pH 7.8, 250 mM NaCl, 5% glycerol and 200 mM imidazole). Eluted proteins were loaded onto a Hi-Load 26/60 superdex-75 size-exclusion column in 100 mM Tris HCl (pH 7.8) buffer containing 50 mM NaCl and 5% glycerol. The concentrations of TIMPs were measured with a NanoDrop 1000 spectrophotometer and by titration against MMP13 essentially as described [25, 26].

### Inhibition constant (K_i_^app^) measurement

Binding study was carried out in fluorescence assay buffer (10 mM CaCl_2_, 50 mM Tris HCl pH 7.5, 0.05% Brij-35, 1% DMSO and 0.02% NaN_3_) as reported [26]. Enzyme activity was measured at 37°C with a Biotek Synergy HT microplate reader equipped with a 320 nm excitation and 405 nm emission filters. *K*_i_^app^ values were calculated by plotting the steady-state rates against TIMP concentrations with GraphPad Prism software using the Morrison equation [27]:

$$V_{s}=\;\left(\frac{V_{0}}{2E_{t}}\right)\;x\;\left[\left(E_{t}-\;I_{t}-\;K_{i}^{app}\right)+{\left[{\left(K_{i}^{app}+\;I_{t}-\;E_{t}\;\right)}^{2}+4\;E_{t}\;K_{i}^{app}\right]}^{1/2}\right]$$

where *V*_0_ denotes the rate of reaction in the absence of inhibitor, *E*_*t*_ is the total enzyme concentration, and *I*_*t*_ is the total inhibitor concentration. All kinetic measurements were performed at least twice to confirm the reproducibility of the findings.

### TNF-α and heparin-binding epidermal growth factor alkaline phosphatase (HB-EGF-AP) shedding assays

TNF-α and HB-EGF-AP shedding assays were carried out on cells cultured in low serum Dulbecco's Modified Eagle's Medium (DMEM) that contained 0.5% fetal calf serum, 4.5 g l^-1^ glucose, 100 I.U. ml^-1^ penicillin and 100 μg ml^-1^ streptomycin. The same medium was used for all ensuing shedding, cell proliferation, migration and soft agar assays. For TNF-α shedding assay, cells cultured in 24-well plate were first transfected with 0.1 μg/well of pro-TNF-α cDNA in pcDNA3.1 vector for three days before TIMPs were added to 1, 5 and 10 nM in triplicate. Incubation was continued for another 24 or 48 hours prior to induction with 100 ng ml^-1^ phorbol 12-myristate 13-acetate (PMA) for 3 hours. All ELISA experiments were performed at least three times to confirm the reproducibility of the results.

The procedure for HB-EGF-AP assay was similar to that of TNF-α. Cells pre-transfected with 0.1 μg/well of HB-EGF-AP cDNA were gently washed twice before TIMPs were added to 1, 5, 10 and 20 nM. Incubation was allowed for 2 hours before PMA was added to 100 ng ml^-1^ to induce shedding. Cell medium was harvested 3 hours post-induction for colorimetric assay with the alkaline phosphatase substrate 4-nitrophenyl phosphate and diethanolamine buffer as described [28].

### Effects of TIMPs on cell proliferation

Cells were seeded in equal number (depending on the cell type, typically 0.5-5x10^5^ cells/well) on 12-well plates and incubated overnight at 37°C to allow settlement. Following day, the medium was replaced with fresh DMEM containing TIMPs ranging from 0.5 nM to 50 nM. Incubation was allowed for 2 days before the cells were gently washed, trypsinised and counted with a Countess automated cell counter (Invitrogen).

### Scratch wound healing assay

The protocol for scratch wound healing assay was derived from the one published [29]. Images of the cells were captured with a Nikons Eclipse Ti-S inverted microscope and the mean distance travelled represents the average reading from at least six independent experiments.

### Soft agar colony formation assay

The experiment was performed in 24-well plates that contained a 0.3 ml thick agarose (0.6%) cushion topped by a 0.15 ml thin agarose (0.3%) upper layer in which cancer cells and TIMPs were embedded [30]. The upper layer was in turn covered by 0.3 ml DMEM medium that also contained the same concentration of TIMPs. To make sure that TIMPs were in supply throughout the lengthy incubation period, the medium was replaced once a week with new DMEM containing fresh TIMPs. The assay was set up in triplicate to ensure that the results are reproducible. Colonies larger than 30μm in diameter were counted regularly with a Nikons Eclipse Ti-S inverted microscope over a 30-day incubation period.

### Statistical analysis

Student's t-test was performed using the online calculator in the statistical analysis website www.socscistatistics.com following a two-tailed hypothesis in order to determine the statistical significance (*P* < 0.05 or 0.01, depending on circumstances) between two samples.

## Results

### Expression and purification of T1:TX chimeras in Sf-9 insect cell

**Fig 1:**
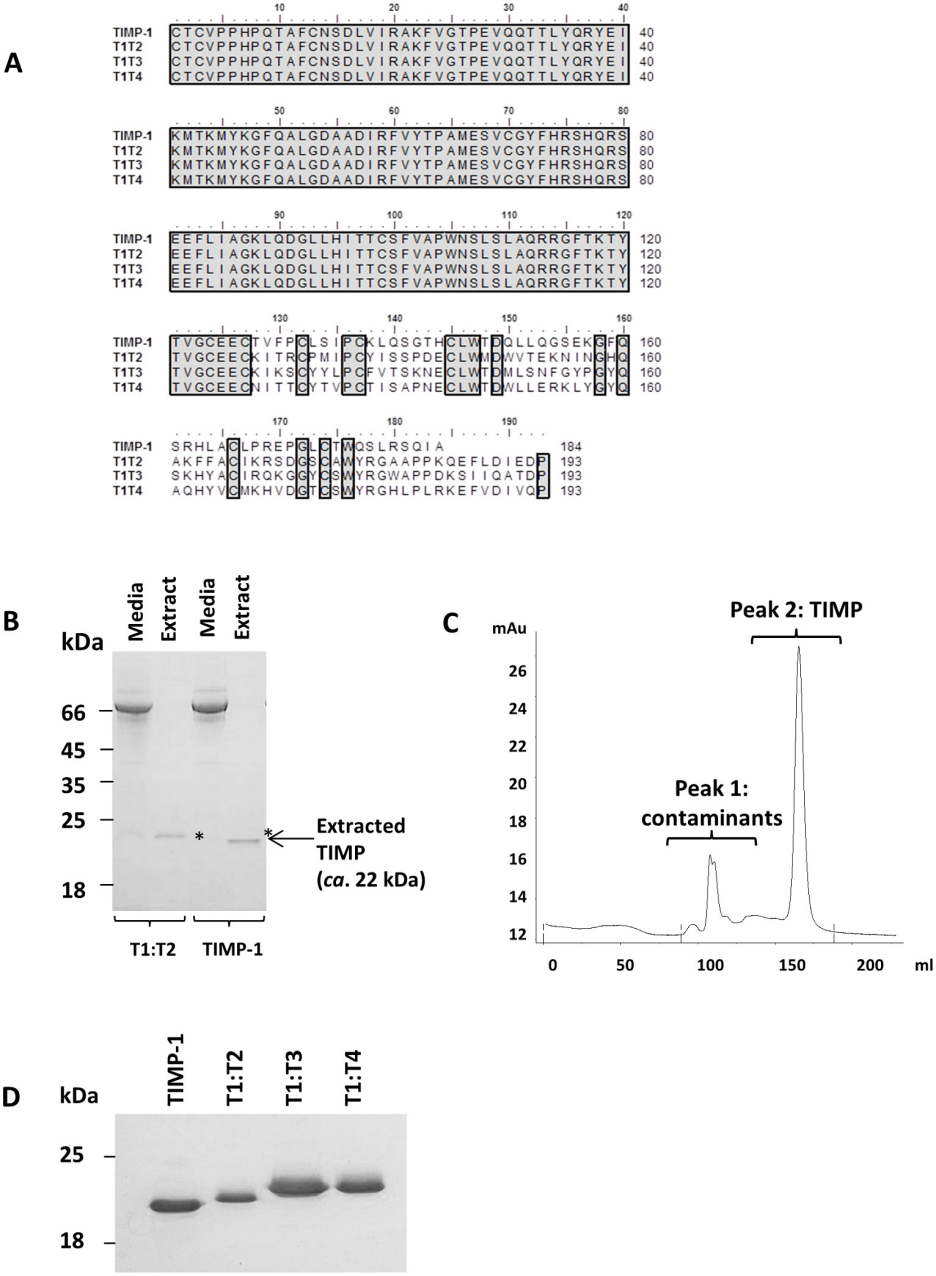
Sequence alignment and purification of T1:TX chimeras using baculovirus/Sf-9 insect cell expression system. (**A**) Sequence comparison of wild type TIMP-1 with T1:T2, T1:T3 and T1:T4 chimeras. Residues Asn30 and Asn78 originally found on wild type TIMP-1 have been mutated to glutamine to prevent undesirable glycosylation by Sf-9 insect cells. (**B**) Examples of the TIMPs (highlighted by asterisks *) extracted from Sf-9 insect cell medium using nickel sepharose excel affinity resin. (**C**) Hi-Load 26/60 superdex chromatogram showing separation of T1:T2 from insect cell proteins. TIMPs (peak 2) typically eluted at 160-170ml, much later than the insect cell proteins (peak 1) (**D**) Homogenous T1:TX chimera proteins on a 12% reducing SDS-PAGE.

Despite two Asn-to-Gln mutations, TIMP-1 and the chimeras were expressed to a high level in baculovirus-infected Sf-9 cell (Fig 1A). Like wild type TIMP-1, T1:TXs were secreted into the medium. The incorporation of an 8x histidine C-terminal tag has allowed the TIMPs to be purified to homogeneity using a simple, two-step purification scheme as shown in Fig 1B–1D. Depending on the constructs, the yield of TIMPs typically ranged from 5–10 mg per-liter of medium.

### Replacement of the C-terminal domain of TIMP-1 by the C-termini of TIMP-2/-3/-4 improves its affinity for ADAM10, ADAM17, MMP14 and MMP19

**Fig 2:**
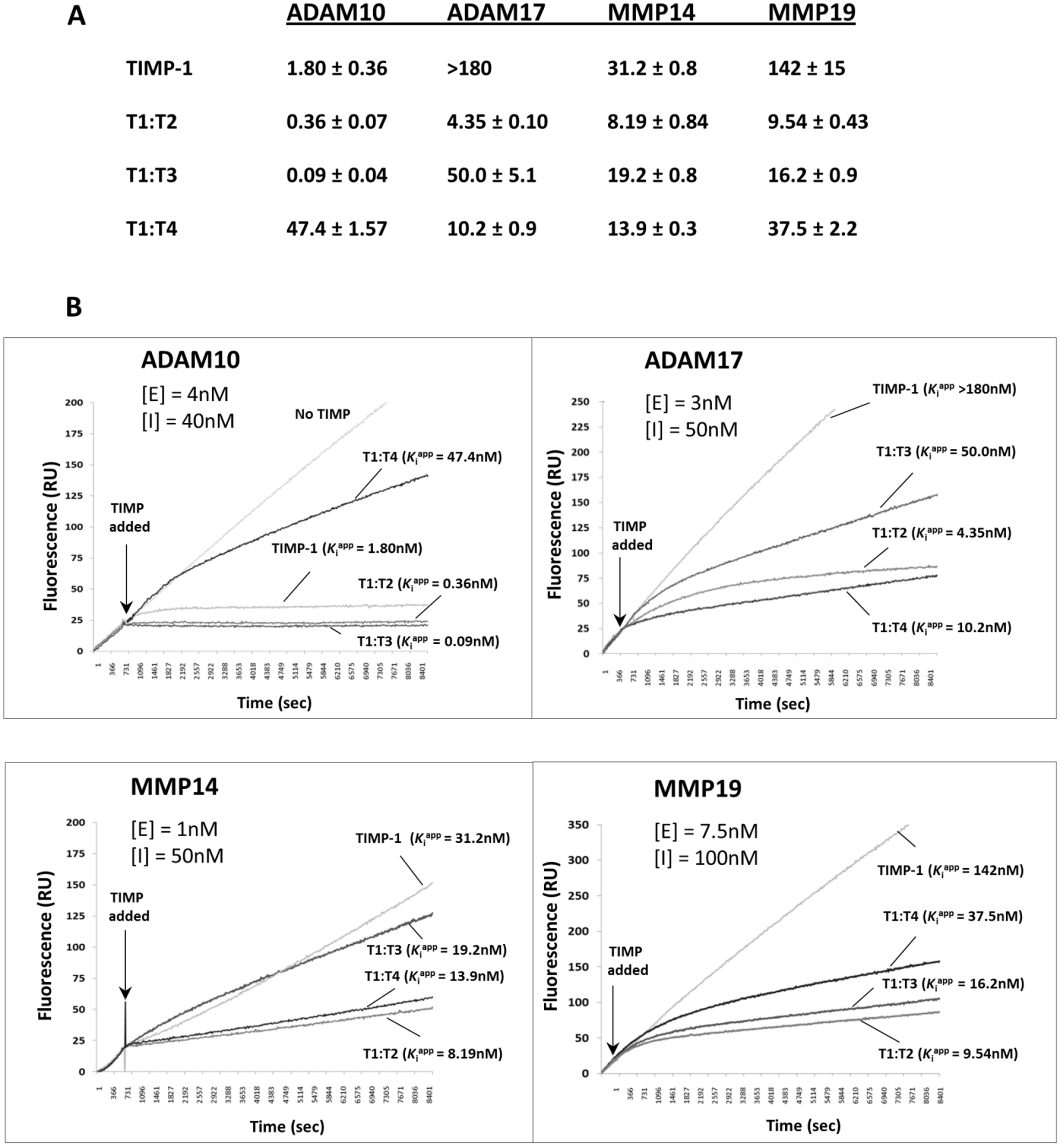
Inhibition profiles of ADAM10, ADAM17, MMP14 and MMP19 by T1:TX chimeras. (**A**) Apparent binding constants (*K*_i_^app^) of TIMP-1 and T1:T2, T1:T3, T1:T4 chimeras for ADAM10, ADAM17, MMP14 and MMP19. Replacement of the C-terminal domain of TIMP-1 by the C-termini of TIMP-2, -3 and -4 dramatically increased the affinity of the TIMP for some metalloproteases. The existing affinity of TIMP-1 for ADAM10 was also significantly improved by the replacement of its C-terminal domain by the C-termini of TIMP-2 and -3. (**B**) The association curves of T1:TX chimeras against the ADAMs and MMPs. In the majority of cases, T1:TXs were significantly more active than wild type TIMP-1. [E] = concentration of enzyme; [I] = concentration of TIMPs.

Fig 2A summarises the *K*_i_^app^ values of TIMP-1 and the chimeras against ADAM10, ADAM17, MMP14 and MMP19 as measured in our laboratory. While the affinity of T1:T2 for ADAM10 (*K*_i_^app^ 0.36 nM) was a notable improvement compared to TIMP-1 (*K*_i_^app^ 1.8 nM), T1:T3 was even more impressive (*K*_i_^app^ 0.09 nM). In truth, T1:T3 was able to establish a tight stoichiometric complex with ADAM10 instantaneously upon encountering the enzyme. On the other hand, the C-terminal domain of TIMP-4 virtually abolished the affinity of TIMP-1 for ADAM10 (*K*_i_^app^ T1:T4 >47 nM). The association curves illustrating the respective potency of the TIMPs are shown in Fig 2B.

We next subjected ADAM17 to the same assay. In agreement with previous report, TIMP-1 has negligible activity on ADAM17 (*K*_i_^app^ >180 nM). In contrast, a varying degree of potency was noted among T1:TXs with the most active chimera being T1:T2 (*K*_i_^app^ 4.3 nM) followed by T1:T4 (*K*_i_^app^ 10.2 nM) and T1:T3 (*K*_i_^app^ 50 nM). With an impressive *K*_i_^app^ value of <5 nM, the affinity of T1:T2 was a significant improvement compared to TIMP-1. The transformation of TIMP-1 from an inactive to a slow, tight-binding inhibitor against ADAM17 is best summarized by the association curves in Fig 2B.

MMP14 has a completely different profile of TIMP reactivity as the enzyme is sensitive to most of the TIMPs with the exception of TIMP-1. In this study, we found that the affinities of T1:T2 and T1:T4 for MMP14 (*K*_i_^app^ 8.2 and 13.9 nM respectively) were also a significant improvement in comparison to TIMP-1 (*K*_i_^app^ 31 nM). With a *K*_i_^app^ value of >19 nM, T1:T3 appeared not so different from TIMP-1 in term of its affinity for MMP14.

Unlike other ADAM and MMP subjects in this study, MMP19 is a soluble enzyme. The reason MMP19 was included in this study was because the TIMP sensitivity profile of the enzyme is very similar to MMP14 in that it can be inhibited by TIMP-2, -3 and -4 but not TIMP-1 [23]. As shown in Fig 2, whilst TIMP-1 was barely active against MMP19 (*K*_i_^app^ 142 nM), T1:T2 and T1:T3 exhibited a much higher affinity of 9 nM and 16 nM. T1:T4 was again a mediocre inhibitor with a relatively high *K*_i_^app^ value of just over 37 nM.

### Exogenously added T1:TX proteins inhibit TNF-α and HB-EGF shedding in cell-based environment

We next assessed the activity of T1:TX chimeras against native sheddases in cell-based environment using two independent TNF-α and HB-EGF shedding assays. To identify a cell type that produced high level of sheddases suitable for the purpose of this experiment, seven cell lines were tested for their TNF-α and HB-EGF shedding ability, namely: A549 lung adenocarcinoma, human embryonic kidney cell-derivative AAV293, BHK21 fibroblast, cervical carcinoma HeLa, HT1080 fibrosarcoma, MCF7 breast cancer cell and the cervical cancer cell SiHa. The cells were transiently transfected with an equal amount of pro-TNF-α or HB-EGF-AP cDNAs and the levels of released TNF-α and HB-EGF in the medium measured by ELISA or colorimetric assay using established protocols outlined under **Experimental Procedures**.

**Fig 3:**
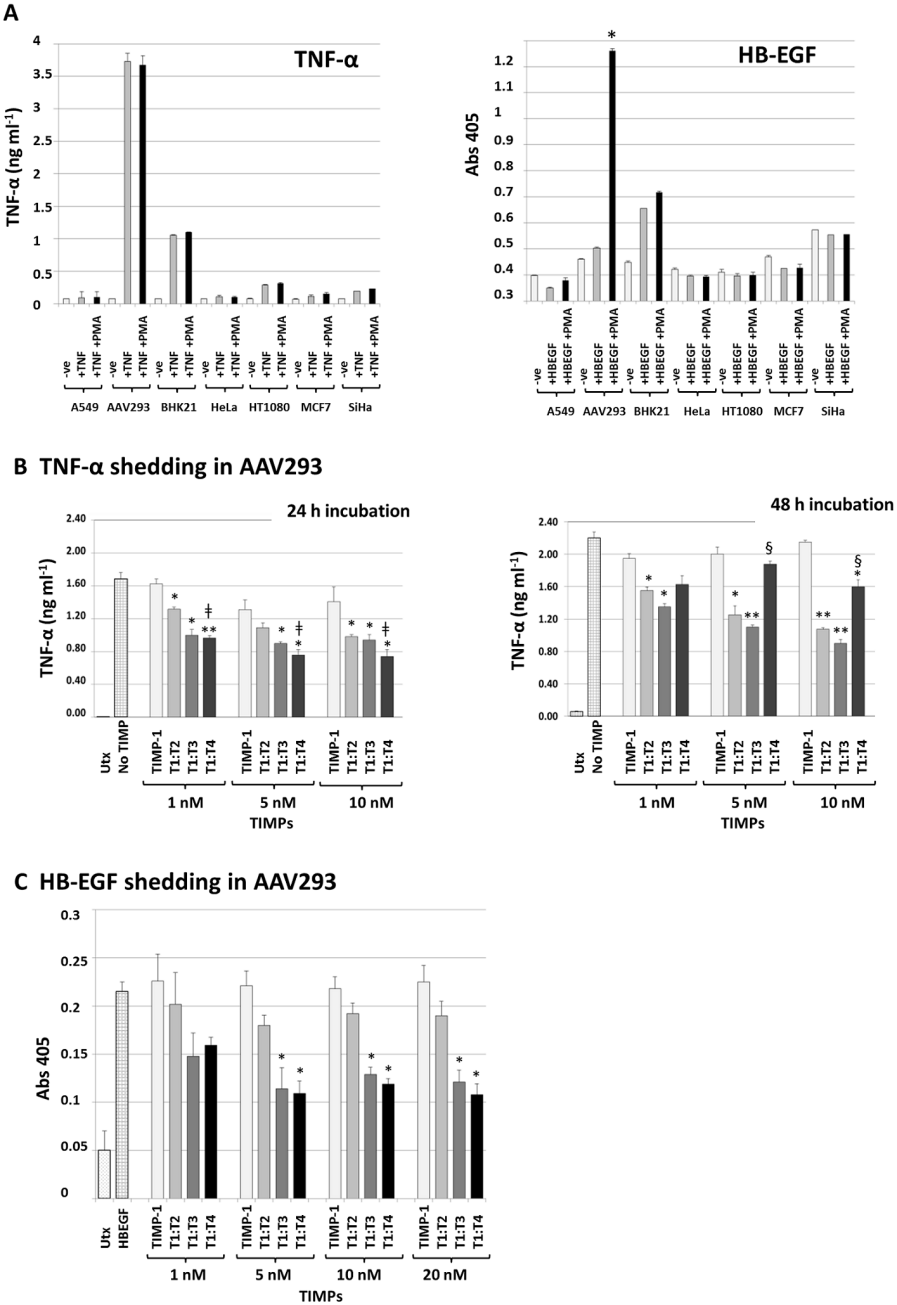
T1:TXs inhibit TNF-α and HB-EGF shedding in cell-based environment. (**A**) AAV293 consistently shed more TNF-α and HB-EGF than other cell types possibly because of its ease of transfection. Note that in TNF-α, there was no difference between cells stimulated or unstimulated with 100 ng ml^-1^ PMA whereas the level of shed HB-EGF was much higher in AAV293 stimulated with PMA (*P < 0.01 compared to without PMA). (**B**) TNF-α shedding assay: relative potency of T1:TXs at 24- and 48-hour incubation time points. T1:TXs inhibit TNF-α shedding significantly better than TIMP-1 (*P < 0.05, ** *P* < 0.01 compared to TIMP-1; left and right). While T1:T4 was more active than T1:T2 (^**ǂ**^ *P* < 0.05; left) at 24-hour incubation time point, the chimera was outperformed by T1:T2 and T1:T3 after 48-hour (^§^ *P* < 0.05; right) (**C**) HB-EGF shedding assay: again T1:TXs were more potent than TIMP-1 (*P < 0.05). UTx = untransfected cells.

As shown in Fig 3A, AAV293 exhibited the highest levels of shedding activities for both TNF-α and HB-EGF, possibly because of the ease of transfection of this cell line. In the case of TNF-α, no significant difference was observed between PMA-stimulated and unstimulated cells. The level of shed HB-EGF in AAV293, however, was several folds higher in cell stimulated with PMA than unstimulated cell (*P < 0.01). Given the outstanding performance of AAV293 in both instances, the cell line was chosen for the ensuing TNF-α and HB-EGF shedding assays.

In view of the already high shedding capability of AAV293, TNF-α shedding assay was carried out with no PMA stimulation. Fig 3B shows that, whilst TIMP-1 lacked the ability to suppress TNF-α release even at 10 nM, T1:TXs were able to reduce shedding by as much as 40% (*P < 0.05, **P < 0.01 T1:TXs compared to TIMP-1 in Fig 3B left and right). Among the chimeras, T1:T4 was marginally more active than T1:T2 (^**ǂ**^ *P* < 0.05; Fig 3B left) and possibly T1:T3 at 24-hour incubation time point. A different outcome emerged if the incubation period was extended to 48 hours. Instead, T1:T4 would be outperformed by T1:T2 and T1:T3 by a significant margin (^§^ *P* < 0.05 at 5 and 10 nM TIMPs; Fig 3B right), an indication that the effect of the chimera could be less long-lasting than its fellow TIMP counterparts possibly due to the instability of the chimera under the assay condition.

In contrast to TNF-α assay, HB-EGF shedding experiments were performed in the presence of 100 ng ml^-1^ of PMA. In total, four doses of TIMPs (1, 5, 10 and 20 nM) were tested for their ability to inhibit HB-EGF shedding in AAV293 cell transiently expressing the ligand. As was found in previous assay, TIMP-1 showed no sign of potency even at 20 nM. Among the chimeras, T1:T3 and T1:T4 outperformed T1:T2 by a large margin; the duo was able to reduce HB-EGF shedding by almost 50% (*P < 0.05; Fig 3C). Comparing the results with the outcome from TNF-α assay, the profile of TIMP activity was not exactly the same suggesting that the sheddases involved in the two experiments may not be completely identical.

### Unlike TIMP-1, T1:TXs have no growth-promoting activity

**Fig 4:**
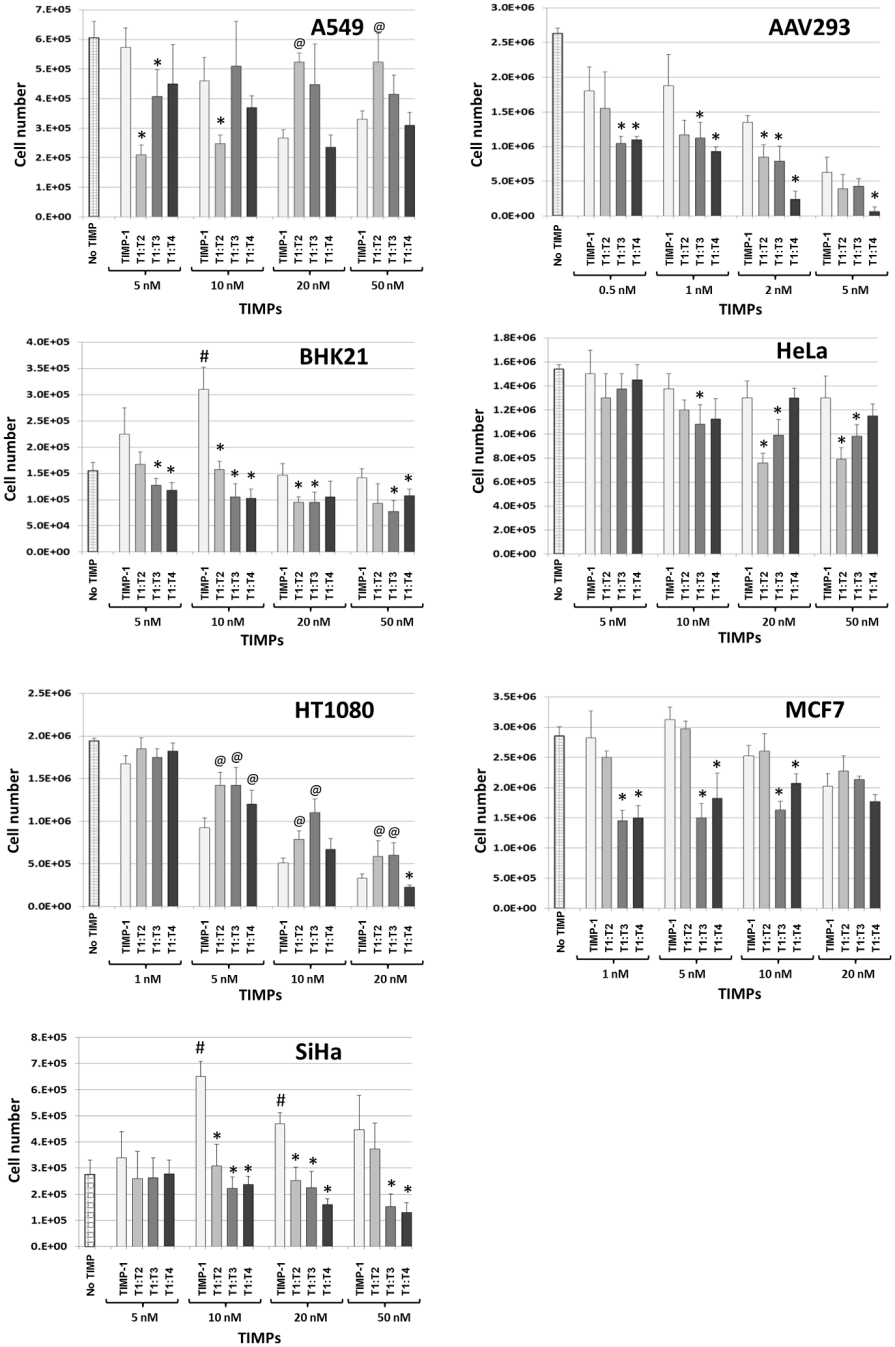
Unlike TIMP-1, T1:TX chimeras lack growth-promoting activity. TIMP-1 had a stimulatory effect at low doses and an inhibitory effect at higher doses on BHK21 and SiHa. The stimulatory activity of TIMP-1 on BHK21 and SiHa at low concentrations can be clearly discerned from the bar charts (^#^ *P* < 0.05 compared with no TIMP). In contrast, the impacts of T1:TXs were mostly inhibitory. While the inhibitory activity of T1:TXs on AAV293 and HT1080 was clearly a linear, dosage-dependent one, increasing concentration of the TIMPs had a lesser impact on A549, BHK21 and MCF7. Note that in AAV293, BHK21 and SiHa, T1:TXs were noticeably more potent than TIMP-1 across a broad range of concentrations (*P < 0.05 compared with TIMP-1 samples). Interestingly, T1:TXs appeared to be less effective than TIMP-1 in suppressing cell growth in HT1080 and A549 at concentration above 20 nM (^@^ *P* < 0.05 compared to TIMP-1 samples of the same concentration).

A major concern that has long overshadowed the therapeutic potential of TIMP-1 is the ability of the inhibitor to act as a growth-promoting factor at low doses (reviewed in [21]). Thus, one of the priorities of this study was to determine if any of the T1:TX chimeras exhibit such potentiation effect and if so, in which cell lines. In this project, we found that TIMP-1 promoted BHK21 and SiHa cell growth at low doses but not in A549, AAV293, HeLa, HT1080 and MCF7 (Fig 4). In comparison, none of the T1:TX chimeras exhibited such potentiation activity.

Fig 4 compares the growth-promoting activity of TIMP-1 and T1:TXs alongside each other. For A549, TIMP-1 had a slight inhibitory impact on cell growth as the concentration was raised from 5 to 50 nM. T1:T2 displayed an exact opposite effect as it appeared to suppress cell growth at 5 nM (*P < 0.05 vs. TIMP-1) but the effect was ameliorated by a further increase in concentration to 50 nM. T1:T3 was more mundane, it had neither a stimulatory nor an inhibitory impact on the cell. Indeed we failed to observe any difference between cells treated and untreated with the chimera. The effect of T1:T4 was nearly a mirror image of that of T1:T2 except that the dosage required for inhibition was nearly four-fold higher than that of T1:T2 (20 nM).

**Fig 5:**
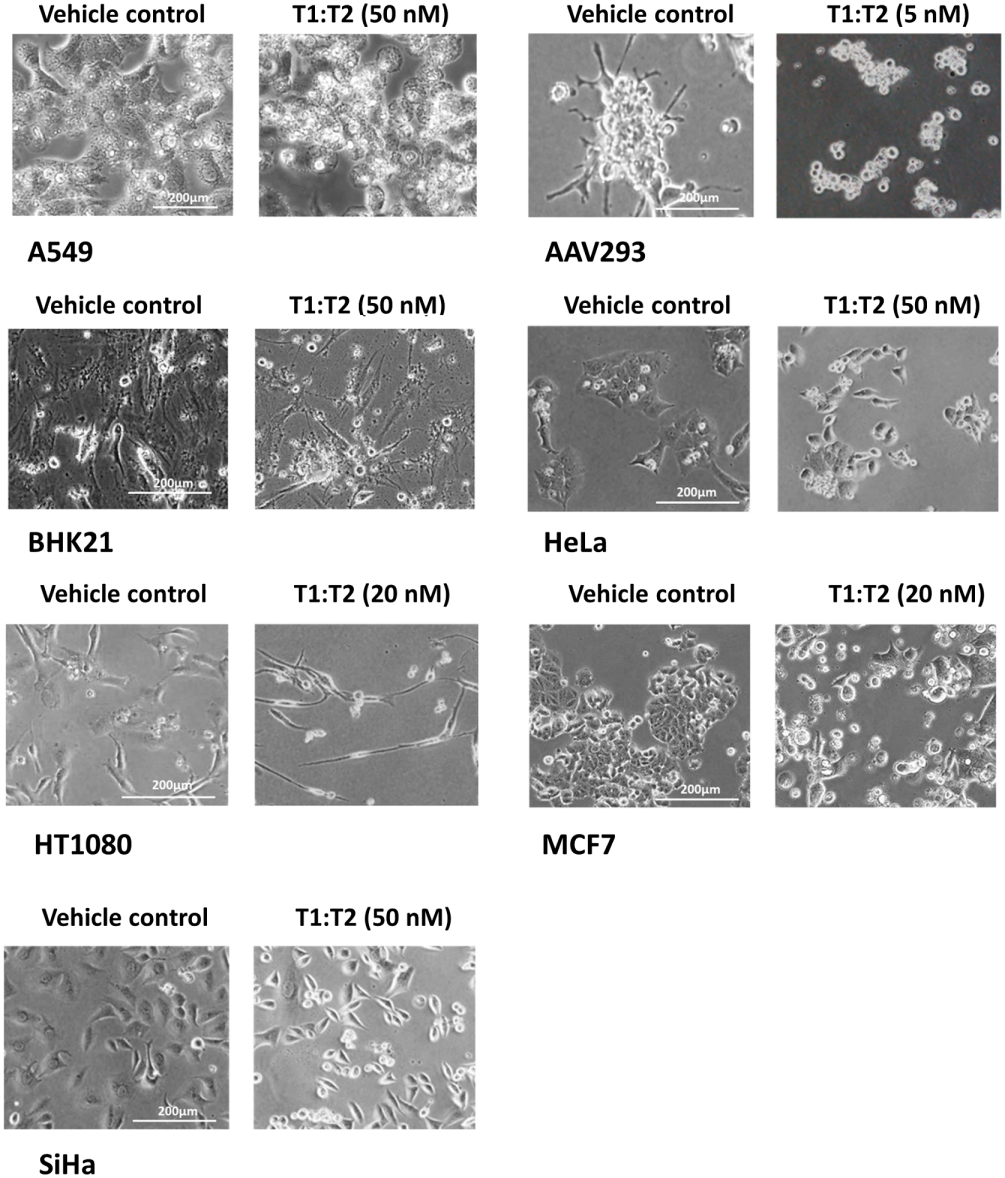
Prolonged exposure to the TIMPs leads to damages in invadopodia/lamellipodia protrusions in AAV293 and HT1080 cells: exemplary pictures after 4 days’ exposure to T1:T4. TIMPs, in particular T1:T4, inhibited invadopodia/lamellipodia formation in several cancer cell lines. AAV293 was the most sensitive among the cells tested, exposure to 5 nM of T1:T4 for over 4 days was sufficient to cause a total loss in cell adhesion. HT1080 was also sensitive to the action of the TIMPs but the concentration required to achieve the same effect was significantly higher (>20 nM).

The response of AAV293 was the most dramatic one. Exposure to the TIMPs as low as 2 nM for over four days often led to damages in invadopodia/lamellipodia protrusions and cell shrinkage that eventually resulted in a total loss of cell adhesion (exemplary picture in Fig 5). The same effect can be achieved in a shorter period by increasing the TIMP concentration to over 10 nM. In this study, we strived to conduct cell counting within two days of exposure to the TIMPs before cell detachment occurred. Fig 4 shows that, without exception, T1:TXs were more effective than TIMP-1 in suppressing cell growth (*P < 0.05). T1:T4 in particular, had the ability to inhibit invadopodia/lamellipodia formation even at very low doses. The effect of T1:T4 became even more pronounced as the concentration reached 5 nM. Further increase in dosage resulted in massive cell detachment from the culture dish and quantification of inhibition was no longer reliable (result not shown).

BHK21 was very tolerant of the TIMPs. The cell displayed no change in morphology or growth rate even in 50 nM of T1:TXs. Fig 4 also shows that TIMP-1 had a rather modest stimulatory impact on BHK21 at 10 nM (^#^ *P* < 0.05) and the same effect was not detected in T1:TXs. Among the chimeras, there was no real difference in cell reaction between 20 to 50 nM of the TIMPs.

HeLa was perhaps the least responsive among the cell lines chosen. Neither TIMP-1 nor T1:TXs appeared to have much impact on its growth rate or morphology even in the high dose of 50 nM. T1:T2 however, did show a better growth-suppressing activity than the other chimeras but only by a small margin (*P < 0.05 T1:T2 vs. TIMP-1 at 20 and 50 nM).

The response of HT1080 was a linear, dose-dependent one similar to that of AAV293. Though not as sensitive as AAV293, exposure to 20 nM of TIMPs completely halted the ability of the cell to multiply. Furthermore, the cell also underwent transformation into an unusually elongated and slender shape unseen in normal healthy cell (Fig 5). A unique observation we noted in HT080 was that T1:TXs were not as active as TIMP-1 as a growth suppressor (^@^ *P* < 0.05). Indeed, HT1080 was the only cell type where T1:TXs were outperformed by TIMP-1.

The reaction of MCF7 mirrored that of BHK21 except that TIMP-1 had no stimulatory ability on the cell. At doses below 10 nM, T1:T3 and T1:T4 were significantly more potent than TIMP-1 and T1:T2 (*P < 0.05) but as the TIMP concentration reached 20 nM, the inhibitory effect of T1:TXs became indistinguishable from that of TIMP-1.

Of the seven cell lines investigated, only two showed sign of stimulation by TIMP-1. Besides BHK21, SiHa was the second cell that responded positively to TIMP-1. Indeed, the distinction between the stimulatory and inhibitory actions of TIMP-1 was the most obvious in this cell. TIMP-1 continued to stimulate SiHa’s growth even at concentration as high as 20 nM (^#^ *P* < 0.05 compared to no TIMP). In contrast, none of the T1:TX chimeras displayed such cell potentiation ability.

### T1:TXs inhibit cell migration in scratch wound healing assays

**Fig 6:**
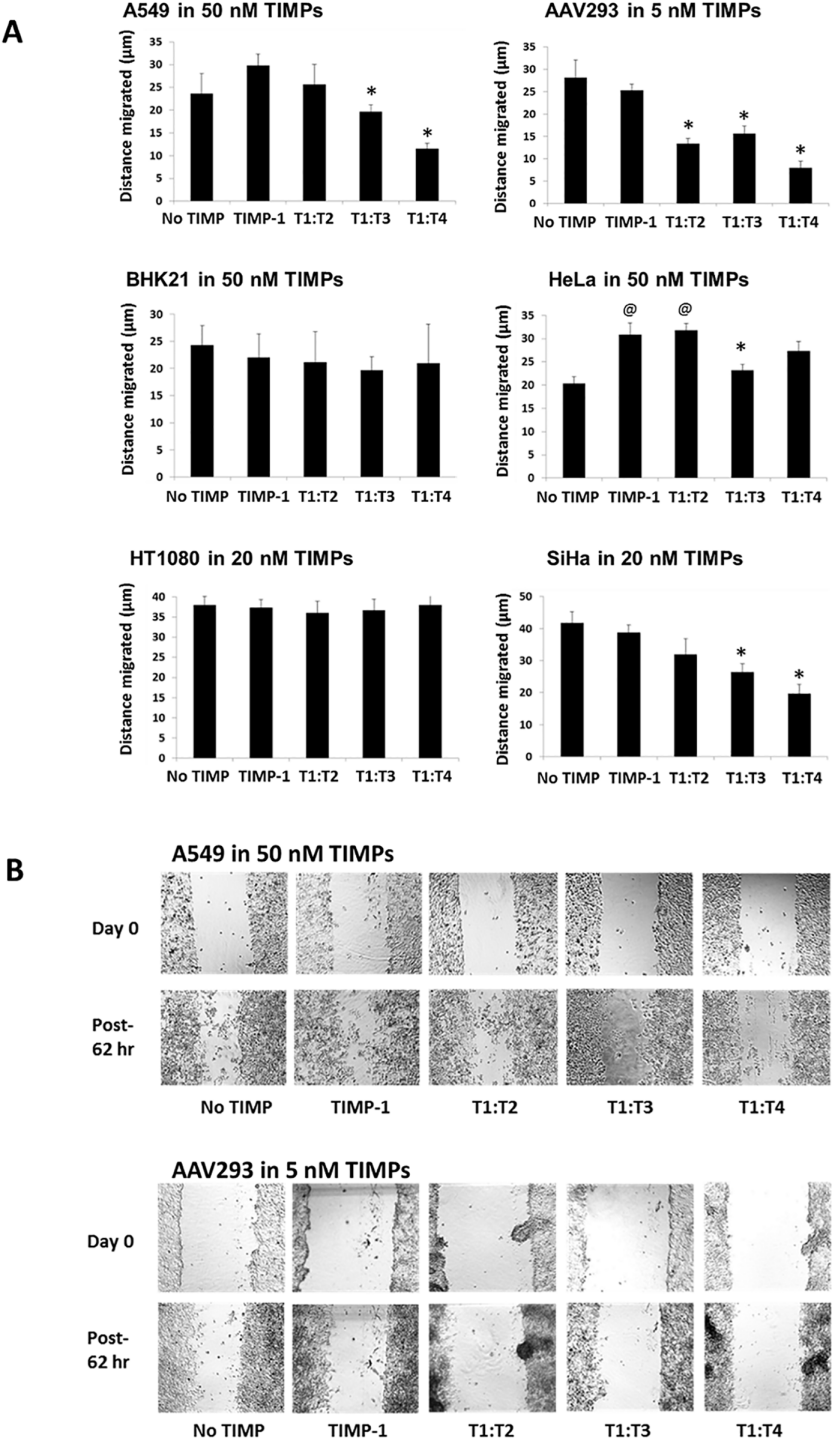
Effect of T1:TX chimeras on cell migration in scratch wound assay: a comparative study with TIMP-1. (**A**) Relative potency of T1:TXs on cell migration. Whilst BHK21 and HT1080 were largely unaffected by the TIMPs, AAV293 was very sensitive to T1:T2 and T1:T4. Among the chimeras, T1:T3 and T1:T4 were the most efficacious in blocking cell migration in A549, AAV293 and SiHa (compared to TIMP-1, *P < 0.05). (**B**) Exemplary pictures showing the impact of the TIMPs on A549 and AAV293 migration.

Based on observation from nine independent but essentially reproducible studies, we can classify the cells into three categories. The first category was composed of cells that responded to the inhibitory action of T1:TXs, namely A549, AAV293 and SiHa. In general, the cells were not responsive to wild type TIMP-1 but to some of the chimeras, in particular T1:T4. At concentrations between 20 and 50 nM (or 5 nM for AAV293), T1:T4 hindered the migration of these cells 40–60% better than TIMP-1 (*P < 0.05; Fig 6A and 6B). T1:T2 was also highly effective in slowing down AAV293 but less so in A549 and SiHa.

The second category was made up of BHK21 and HT1080. The cells were entirely insensitive to the TIMPs at concentration as high as 50 nM. Neither TIMP-1 nor T1:TXs had any noticeable impact on the cells’ migration. In the final category was HeLa. Instead of being inhibited by TIMPs, the cell was stimulated by TIMP-1 and T1:T2 (^@^ *P* < 0.05 vs. no TIMP).

MCF7 was the least metastatic among the cells chosen. The cell was not included in Fig 6 as it showed negligible sign of migration even after one week of incubation under identical culture condition.

### Exogenously added T1:TX proteins inhibit colony formation in soft agarose matrix

**Fig 7:**
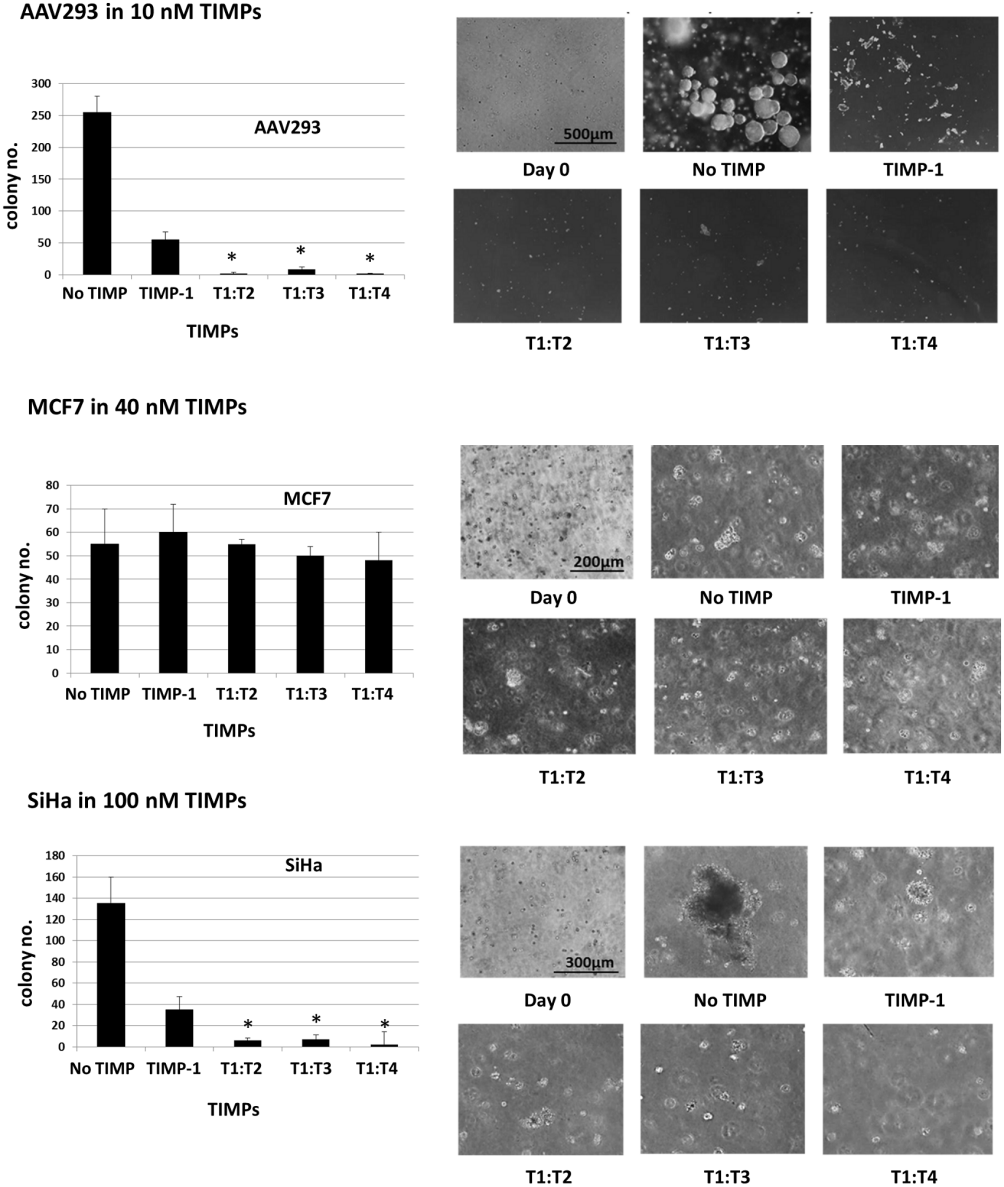
Clonogenic assay: T1:TX chimeras inhibit AAV293 and SiHa colony formation in soft agarose matrix. T1:T4 was the most effective in hampering colony development followed by T1:T2 and T1:T3 (*P < 0.05 compared to TIMP-1). In contrast, MCF7 colony formation did not appear to be affected by the TIMPs.

Our next aim was to find out if the potency of T1:TXs, in particular T1:T4, observed in two-dimensional (2D) cell culture could be replicated in a three-dimensional (3D) environment. Between the selected cells, only AAV293, MCF7 and SiHa were able to develop into multi-cellular colonies of substantial size after an incubation period of 3–4 weeks. Without exogenously added TIMPs, AAV293 quickly developed into large, multi-cellular colonies ranging between 30 to 250 μm in diameter. Inclusion of 10 nM TIMP-1 in the matrix drastically stunted colony formation but its efficacy was nowhere as impressive as that of T1:T4. In agarose that contained 10 nM of T1:T4, there was nearly no sign of cell growth (*P < 0.05 T1:T4 compared to TIMP-1). T1:T2 and T1:T3 were also more effective than TIMP-1 though not as potent as T1:T4 (Fig 7).

The reaction of SiHa was very similar to that of AAV293 in that the cell can be most effectively inhibited by T1:T4 closely followed by T1:T2 and T1:T3. TIMP-1 did impact negatively on the colony formation but the effect was no way as pronounced as the other chimeras. Although MCF7 was able to grow into multi-cellular colonies, the cell did not appear to be significantly affected by the TIMPs. On the other hand, A549, BHK21, HeLa and HT1080 cells grew at a very slow pace under identical culture condition. Their colonies were too small to allow accurate judgment to be made even after 30 days of incubation (results not shown).

Based on the data collected from AAV293 and SiHa cells, we can conclude that the pattern of TIMP inhibition in the 3D environment was in agreement with that of the 2D culture.

## Discussion

Our findings are significant for several reasons. Firstly, attempts to engineer TIMPs for MP targeting have so far focused on the N-terminal domain despite kinetic and crystallographic evidence suggesting that the C-terminal domain is also involved in MP interaction [31–33]. Here, we demonstrate that the C-terminal domain of the TIMPs can also be exploited for engineering. A revelation that we find most perplexing is that the affinity of a T1:TX chimera for an individual MP cannot be predicted from the inhibition profile of the wild type TIMP parents from which the chimera is derived. This finding is best exemplified by the affinity of T1:T2 chimera for ADAM10. Even though wild type TIMP-2 is a known poor inhibitor for ADAM10 [34], transplantation of its C-terminus to TIMP-1 (i.e. T1:T2) transformed the TIMP into a superb inhibitor for the ADAM with an affinity (*K*_i_^app^ 0.36 nM) surpasses even that of TIMP-1 (*K*_i_^app^ 1.80 nM). At present, we are unclear as to how the C-terminal domain interacts with the ADAMs. We are currently setting up T1:T2/ADAM10 co-crystallisation trials in the hope of elucidating the underlying mechanism.

Furthermore, we have shown here the superiority of T1:TX chimeras over wild type TIMP-1 in the blocking of membrane-anchored sheddases. The main sheddase for TNF-α is ADAM17 whilst HB-EGF shedding is carried out by a broader range of ADAMs including ADAM9, -10, -12, -17 and -19 [5, 28, 35, 36]. Interestingly, comparison of the kinetic information in Fig 2 with the cell proliferation assay outcomes (Figs 4, 6 and 7) reveals that the cell-suppressing activity of T1:TXs has very little relation with the affinities of the TIMPs. Based solely on the kinetic data, one would expect T1:T2 to outperform T1:T4 as T1:T2 has a much superior affinity for all the ADAMs and MMPs tested. Instead, T1:T4 consistently surpassed the other chimeras in the majority of the cell-based proliferation assays. The reason, we surmise, lies in the MP-independent functions of the TIMPs. In addition to serving as a MP inhibitor, TIMP-1 is known to promote cell growth and angiogenesis via MP-independent pathways. One of the mechanisms by which TIMP-1 exerts its MP-independent activity is through Wnt/β-catenin signaling in conjunction with the surface receptor CD63 [37, 38]. At present, we do not know if any of the T1:TX chimeras are capable of eliciting such MP-independent activity and if so, to what extent. Neither do we know if the chimeras possess any cytotoxicity effect not present in wild type TIMP-1. Our data do however suggest that T1:TXs have no growth-promoting ability in all the cell-based tests carried out.

Table 1 summarises the various cellular effects of T1:TXs in all the seven immortalised cell lines tested in this project. Throughout this study, we are intrigued by how diverse the cells’ reactions are towards the TIMPs, an example being the contrasting responses in cell growth exhibited by the cells as shown in Fig 4. While T1:TXs were consistently more potent than TIMP-1 in suppressing AAV293, BHK21 and SiHa cell proliferation across a broad range of concentrations, the opposite was true for HT1080. On the contrary, A549 and HeLa exhibited a mixed reaction towards T1:TXs that appears to be dosage-related. At concentration above 10 nM, T1:T2 even became less inhibitory in A549 cells, at least at the concentrations tested. This diversity in reactions was also observed in the subsequent cell migration assay (Fig 6). Among the cell lines that displayed metastatic ability, the inhibitory activity of T1:TXs was more obvious in A549, AAV293 and SiHa and less so in BHK21 and HT1080. The seven cell lines included in this study were selected at the outset of this project for their different origin and metastatic potential. In particular, AAV293 and HT1080 were chosen for their pronounced invadopodia and lamellipodia protrusions in which the membrane-anchored MMP14 is known to be sequestered [8, 39]. Here, we doubt if the cellular potency of the TIMPs to inhibit these protrusions has any link with their MP-inhibiting ability as even TIMP-1 can cause the cells to lose the protrusions. In our opinion, the varied reaction observed reflects the dissimilar intracellular machineries that exist in these cell types. As T1:TX chimeras are non-native, man-made proteins that do not exist in the nature, we have yet to delineate their profiles with regard to MP activation/inhibition, pro-/anti-apoptotic activity or cell potentiation functions in different cell lines. These assessments will be carried out more thoroughly in the next phase of our investigation.

**Table 1:** Summary of the cellular effects of T1:TXs as observed in the seven immortalized cell lines in this project.

**Cell Proliferation Inhibition:**				
	**TIMP-1**	**T1:T2**	**T1:T3**	**T1:T4**
**A549**	**+**	**+ +** ^a^	**0**	**+**
**AAV293**	**+ +**	**+ +**	**+ +**	**+ + +**
**BHK21**	**- -**	**0**	**0**	**0**
**HeLa**	**0**	**+**	**+**	**0**
**HT1080**	**+ +**	**+**	**+**	**+ +**
**MCF7**	**0**	**0**	**+**	**+**
**SiHa**	**- -**	**0**	**+**	**+**
**Cell Metastasis Inhibition:**				
	**TIMP-1**	**T1:T2**	**T1:T3**	**T1:T4**
**A549**	**-**	**0**	**0**	**+ +**
**AAV293**	**0**	**+ +**	**+**	**+ + +**
**BHK21**	**0**	**0**	**0**	**0**
**HeLa**	**- -**	**- -**	**0**	**-**
**HT1080**	**0**	**0**	**0**	**0**
**SiHa**	**0**	**0**	**+**	**+ +**
**3D Colony Formation Inhibition:**				
	**TIMP-1**	**T1:T2**	**T1:T3**	**T1:T4**
**AAV293**	**+**	**+ + +**	**+ +**	**+ + +**
**MCF7**	**0**	**0**	**0**	**0**
**SiHa**	**+**	**+ +**	**+ +**	**+ + +**

(+) inhibitory effect;

(-) stimulatory effect;

(0) no effect observed.

^a^ The inhibitory effect of T1:T2 on A549 was apparent only at concentrations below 20 nM.

We present in this report an important finding that could form the basis of a new tactic in TIMP engineering. Replacement of the C-terminal domain, as we show here, can be an alternative means to alter the potency of TIMP-1 for the MPs. ADAMs and MMPs are enzymes of tremendous medical importance. Selective inhibition of the proteinases could provide a realistic therapeutic strategy for diseases such as rheumatoid arthritis and cancers. At present, we have yet to explore the inhibitory activity of T1:TXs for other MMPs and ADAMs. Furthermore, we do believe the chimeras could be further fine-tuned to enhance their specificity against the MPs including members of the ADAMTS (ADAM with thrombospondin motif) family. We believe the chimeras could also provide an insight into the growth factor-like functions of TIMP-1. More detailed investigations in the future are required to find out the answers.
